# Characteristics of Dental Resin-Based Composites in Leukemia Saliva: An In Vitro Analysis

**DOI:** 10.3390/biomedicines9111618

**Published:** 2021-11-04

**Authors:** Alexandru Mester, Marioara Moldovan, Stanca Cuc, Ciprian Tomuleasa, Sergiu Pasca, Miuta Filip, Andra Piciu, Florin Onisor

**Affiliations:** 1 Department of Oral Health, University of Medicine and Pharmacy “Iuliu Hatieganu”, 400012 Cluj-Napoca, Romania; mester.alexandru@umfcluj.ro; 2 Department of Polymer Composites, Institute of Chemistry “Raluca Ripan”, University Babes-Bolyai, 400294 Cluj-Napoca, Romania; miuta.filip@ubbcluj.ro; 3 Department of Hematology, Institute of Oncology “Ion Chiricuta”, University of Medicine and Pharmacy “Iuliu Hatieganu”, 400012 Cluj-Napoca, Romania; ciprian.tomuleasa@umfcluj.ro (C.T.); pasca.sergiu@elearn.umfcluj.ro (S.P.); 4 Department of Medical Oncology, Institute of Oncology “Ion Chiricuta”, University of Medicine and Pharmacy “Iuliu Hatieganu”, 400012 Cluj-Napoca, Romania; andra.piciu@umfcluj.ro; 5 Department of Maxillofacial Surgery and Implantology, University of Medicine and Pharmacy “Iuliu Hatieganu”, 400012 Cluj-Napoca, Romania; florin.onisor@umfcluj.ro

**Keywords:** leukemia, oral health, saliva, dental composite, resin-based composite

## Abstract

Background: The aim was to analyze, in vitro, four resin based composite systems (RBCs) immersed in saliva of leukemia patients before starting chemotherapy regiments. Material and methods: Saliva was collected from 20 patients (4 healthy patients, 16 leukemia patients). Resin disks were made for each RBC and were immersed in the acute leukemia (acute lymphocytic (ALL), acute myeloid (AML)), chronic leukemia (chronic lymphocytic (CLL), chronic myeloid (CML)), Artificial saliva and Control environment, and maintained for seven days. At the end of the experiment, the characteristics and the effective response of saliva from the studied salivas’ on RBCs was assessed using water sorption, water solubility, residual monomer and scanning electron microscopy (SEM). Data analysis was performed and a *p*-value under 0.05 was considered statistically significant. Results: The behaviour of RBCs in different immersion environments varies according to the characteristics of the RBCs. RBCs with a higher filler ratio have a lower water sorption. The solubility is also deteriorated by the types of organic matrix and filler; the results of solubility being inversely proportional on the scale of negative values compared to sorption values. Chromatograms of residual monomers showed the highest amount of unreacted monomers in ALL and AML, and the Control and artificial saliva environments had the smallest residual monomer peaks. Because of the low number of differences between the experimental conditions, we further considered that there were no important statistical differences between experimental conditions and analysed them as a single group. Conclusion: The influence of saliva on RBCs depends on the type of leukemia; acute leukemia influenced the most RBCs by changing their properties compared to chronic leukemia.

## 1. Introduction

Leukemia represents a malignant hematological disorder characterized by an uncontrolled proliferation of immature cells from blood [1]. According to the cell type and clinical manifestation, in acute leukemia (acute lymphocytic (ALL), acute myeloid (AML)), cells are immature, without function and with rapid proliferation in the bone marrow and may determine a fatality in patients without treatment. On the other side, chronic forms (chronic lymphocytic (CLL), chronic myeloid (CML)) have emerged at a slow rate, manifesting with inadequate proliferation of mature, differentiated cells [1]. The choice of leukemia treatment is influenced by the type (acute, chronic), subtype (lymphocytic, myeloid) and may include chemotherapy, radiotherapy, immunotherapy, bone marrow transplantation or hematopoietic stem cell transplantation [1].

Due to their disease, the oncological treatment in such patients effects the oral mucosa and tissues leading to other complications [1,2,3]. Oral manifestations reported are gingival hyperplasia associated with bleeding gums, periodontitis, xerostomia, carious lesions, temporomandibular joint disfunction, lymphadenopathy, mucositis, oral infections, erosions of mandibular bone and may develop into squamous cell carcinoma [1,2,3]. In some situations, recognizing oral manifestations may be an important factor in detecting leukemia [2]. However, the dental treatment approach varies between cancer centers because of the availability of an oral health specialist in oral oncology, difficulty in implementing and following the established protocol, and preference of traditional treatments over the scientifically proved evidence [4]. The most ideal scenario is that all leukemic patients, before starting hematological treatment, should undergo a complete oral and dental evaluation. After a clear diagnosis and treatment plan, all potential active sources of infection (e.g., advanced cavities, compromised restorations, unrestorable cavities, hopeless teeth, periapical lesions, dental abscess, periodontal lesions) should be firstly removed [3,4].

Resin-based composites represent a simpler and faster material in anterior and posterior restorative dentistry. Besides this major advantage, their major disadvantages consist in limited longevity and possible health risks. Their evaluation methods, such as physico-mechanical properties, polymerization reaction kinetics, antibacterial characteristics, cytotoxicity, represent an essential role in the improvement of RBCs [5,6]. Side-effects (e.g., cytotoxic) of RBCs should be taken into account when selecting a material suitable for dental restorations [6]. The release of residual monomer (unreactioned monomer from the polymer matrix) may influence the vitality of pulp cells causing inflammation of oral mucosa [6]. Residual monomers can interact with human oral cells through absorption and solubility processes of RBC that interact with oral fluids permanently [7,8]. 

When it comes to the use of RBCs in carious lesion, current literature does not provide data about the type of RBCs that should be used in leukemia patients. Therefore, the aim of our research was to analyze, in vitro, the degradation process of resin-based composites by determining the sorption and solubility properties and evaluating the amount of residual monomer in saliva of leukemia patients before starting chemotherapy regiments. This approach reflects the properties of dental composite, as well as certain aspects of the potential side-effects caused by the patient’s pathology. Additionally, the subject addressed has a practical connotation, since dental practitioners should be constantly informed and aware about the properties of dental materials and should personalize the selection of restoration materials in accordance with the complexity of each clinical case [9].

## 2. Material and Methods

### 2.1. Study Population and Saliva Sampling

The study protocol was approved by the hospital ethics committee (contract number 183/25.06.2020). Patients agreed to participate in this study with the use of their medical data for scientific purposes. All patients signed the informed consent. Twenty patients, four who were systematic healthy and sixteen with leukemia (ALL, AML, CLL, CML), non-smoking, without periodontal disease, and a mean age of 55.3 years, were recruited from the Department of Hematology, Institute of Oncology “Ion Chiricuta”, Cluj-Napoca, Romania. For saliva sampling, each patient was seated in a relaxed position, with their head bent forward in order to allow saliva to accumulate in the anterior region of the oral cavity. Firstly, patients were instructed not to eat and drink liquids for 2 h, not to use lipstick/lip balm, chewing gum, before the saliva was collected. Secondly, the patient swallowed, and then saliva was collected in a polypropylene tube; during sampling, the patient was asked to avoid swallowing. After collection, the samples were stored at 4 °C and then were stored at −80 °C until the analysis. 

### 2.2. Resin Based Composite Systems

Our research analyzed the characteristics and the effective response of saliva from leukemia patients on four universal resin-based composites (RBC): Enamel, Gaenial, Evetric and Herculite. These dental materials are light-curable composites used for both anterior and posterior dental restorations; their composition is presented in Table 1.

### 2.3. Preparation of RBCs Sample in Saliva Samples

Five resin disks (*n* = 5) were made for each RBC using a Teflon mold with a diameter of 15 ± 1 mm and 1 mm thickness. The mold was completely filled with the blend. After this procedure, a polyester strip was placed over and covered with a glass slide to remove voids and extrude excess composite resin material. The composite was then light cured through the glass strip for 20 s on 5 points (3 points on the first side and 2 points on the other side of the sample after its removed from the mold). The light unit was held rigidly in place and specimens were light activated with the light guide positioned directly on the glass. We used LED curing light Woodpecker (Guilin Woodpecker Medical Instrument CO., Guilin, China) with a constant intensity of 1000 mW/cm^2^.

### 2.4. Water Sorption and Solubility

Water sorption and water solubility evaluation were performed according to ISO 4049/2000 [10]. Immediately after polymerization, the disks were stored individually in a desiccator at 23 °C until a constant weight (M_1_) of disks was established using an analytical balance (Ohaus Explorer) with an accuracy of 0.001 g. Immediately after M_1_ establishment, the disks were immersed, individually, in falcon tubes which contain different immersion environment (ALL, AML, CLL, CML, Artificial saliva and Control) and maintained at a temperature of 37 °C for 7 days. After 24 h, 48 h, 72 h and 7 days, the disks were removed from the immersion environments and gently dried with absorbent paper. Each disk was weighed only one time in an analytical balance to obtain the M_2_ mass. After this, the disks were placed again in a vacuum desiccator for 3 h, until they kept a constant mass M_3_. Sorption (W_p_) and solubility (W_l_) ratios were calculated for each specimen using the following equations: W_p_ = (M_2_ − M_1_) × 100; W_l_ = (M_1_ − M_3_) × 100.

### 2.5. Residual Monomer

The amount of saliva left after the sorption test, in which the dental material samples were immersed for 7 days and kept at a temperature of 37 °C, was frozen and then lyophilized. The analyzes were performed on a Jasco HPLC chromatograph (Jasco International Co., LTD., Tokyo, Japan) that was equipped with an intelligent pump PU-980, a ternary gradient unit LG-980-02, an intelligent column thermostat CO-2060 Plus, an intelligent detector UV-975, and an injection valve that was equipped with a 20 µL sample loop (Rheodyne, Thermo Fischer Scientific, Waltham, MA, USA). The system was controlled and the experimental data analyzed with the ChromPass software (version v1.7, Jasco International Co., LTD., Tokyo, Japan). Separation was performed on a Lichrosorb RP-C18 column (25 cm × 0.46 cm) at a column temperature of 21 °C. The mobile phase was a mixture of acetonitrile (A, HPLC grade) and water (Milipore ultrapure water) and a gradient was applied according to the following method: 0–15 min, linear gradient 50–80% A; 15–25 min, linear gradient 80–50% A. The flow rate was 0.9 mL/min and the injected volume was always 20 μL. UV detection was performed at 204 nm to monitor the elution of all analytes (TEGDMA UDMA and BisGMA,) because it shows significant absorption at this wavelength. Stock solutions of TEGDMA, UDMA and Bis-GMA reference standards (1 mg/mL) were prepared in acetonitrile and stored at 4 °C. The linearity of the response to the analytes was established with four concentration levels and the regression factors R^2^ were higher than 0.998. The all analyses were performed in triplicates, both for standards and for samples. The residual monomer amount has been determined from the HPLC chromatograms of the extracts and it was calculated as the amount of released monomer depending on the weight of the sample.

### 2.6. Scanning Electron Microscopy Analysis

Scanning electron microscopy (SEM) was used to investigate the structure of the RBCs subjected to sorption and solubility test. The investigation of the samples was performed with the INSPECT S electron microscope of the FEI company (Hillsboro, OR, USA). After 7 days of experiment, the disks were removed from the immersion environments and inserted onto filter paper. Each disk was gently dried by buffering with absorbent paper and vacuum drying; then, mounted on aluminium stubs with the side that came in contact with the glass at the time of polymerization upwards. The specimens were then examined using SEM at high vacuum, with an acceleration voltage of 30 kV. The SEM images were captured at the magnification of 1000×.

### 2.7. Statistical Analysis

Data analysis was performed using R4.0.1. Comparisons between more than two small subgroups (2–3 data points) were assessed using ANOVA as a non-parametric assessment would yield a non-significant *p*-value because of the low number of observations. Normality of the distribution was assessed using Shapiro–Wilk test and histogram visualization. Comparisons between multiple non-normally distributed groups were done using Kruskal–Wallis test. Statistically significant assessments in the Kruskal–Wallis test was further analysed using pairwise Mann–Whitney-Wilcoxon rank sum test with Bonferroni correction. Association between a continuous variable and an ordinal variable with a limited number of values was performed using Kendall’s tau. A *p*-value under 0.05 was considered statistically significant.

## 3. Results

### 3.1. Water Sorption, Water Solubility and Residual Monomer

**Figure 1:**
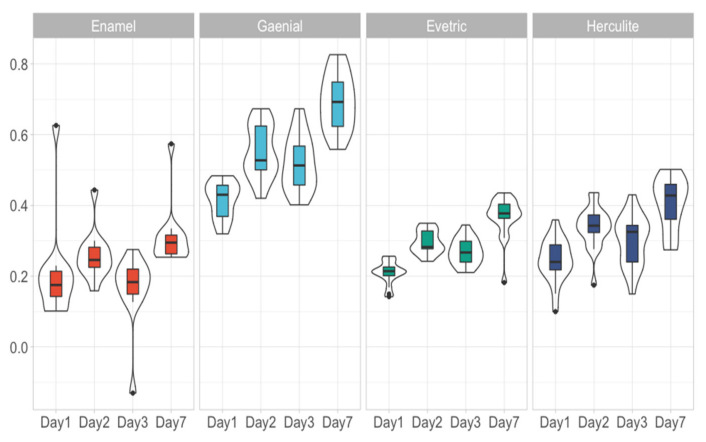
Overview of water sorption measurement considering the material used and the day assessed.

**Figure 2:**
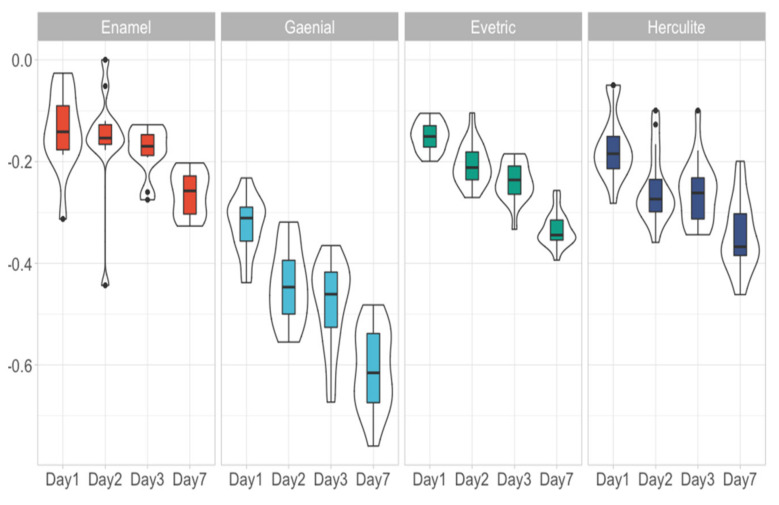
Overview of water solubility measurement considering the material used and the day assessed.

Each material had 18 data points that were followed across 4 days with the exception of Enamel, which had 12 data points that were also followed across Day 1, Day 2, Day 3 and Day 7. These data points were equally divided to ALL, AML, CLL, CML, Artificial saliva and Control. Differences in sorption and solubility between the experimental conditions divided by day, material used were assessed (Table 2). Because of the low number of differences between the experimental conditions, we further considered that there were no important differences between experimental conditions and analysed them as a single group. An overview of the water sorption (Figure 1) and solubility (Figure 2) measurements were presented below. Considering each day there were statistically significant differences between the materials used (*p* < 0.0001). Thus, we analysed the differences between each paired material (Table 3). Added to this, we assessed the progression of each material through the assessment days (Table 4).

**Figure 3:**
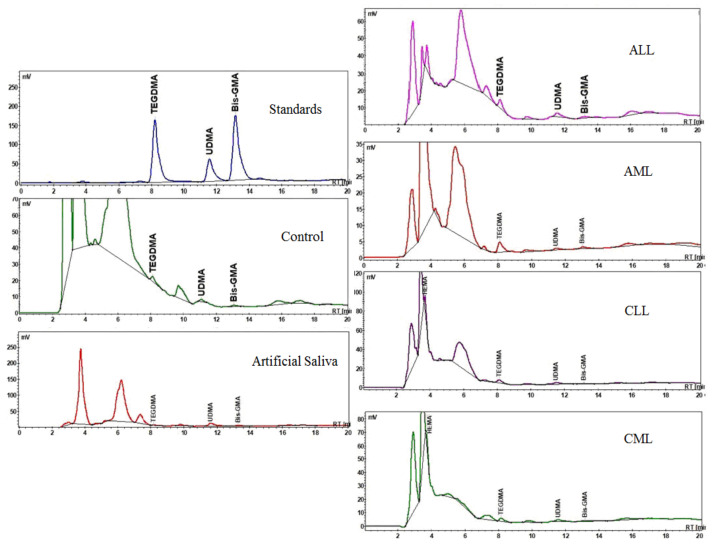
HPLC chromatograms of residual monomers from different saliva immersion.

Resin based composites with a higher filler ratio have a lower water sorption; the increasing sorption from the RBCs are Enamel, Evetric, Herculite and G-aenial. If Enamel shows a value of 0.28 ± 0.024% (depending on the immersion environment) of water in composite on the 7th day of experiment, G-aenial shows a higher value (0.70 ± 0.18%). For Evetric and Herculite RBCs, the sorption values vary very little between the 6 immersion environments, 0.36 ± 0.02% and 0.41 ± 0.07%, respectively. Water sorption of investigated RBCs increases directly proportional with increased immersion time in all 6 environments, their behaviour being similar. The solubility is also deteriorated by the types of organic matrix and filler; the results of solubility being inversely proportional on the scale of negative values compared to sorption values. The highest amount of residual monomers for the RBCs was recorded in the ALL saliva sample, followed by AML, CLL, CML (Table 5, Figure 3).

The HPLC analysis showed individual monomer peaks (TEGDMA, UDMA and Bis-GMA) unreacted in 6 different media after 7 days. The R^2^ values of the standard curves for TEGDMA, UDMA and Bis-GMA are represented graphically in Figure 3. The amount of monomers released in the selected test groups in different media is shown in Table 5. The trend was regularly observed in all groups test for the release of UDMA and TEGDMA monomers in the highest amount released, followed by the Bis-GMA monomer. On the other hand, the groups of composites showed the highest amount of unreacted monomers in ALL and AML, and the Control and artificial saliva environment had the smallest residual monomer peaks.

### 3.2. Scanning Electron Microscopy Analysis

**Figure 4:**
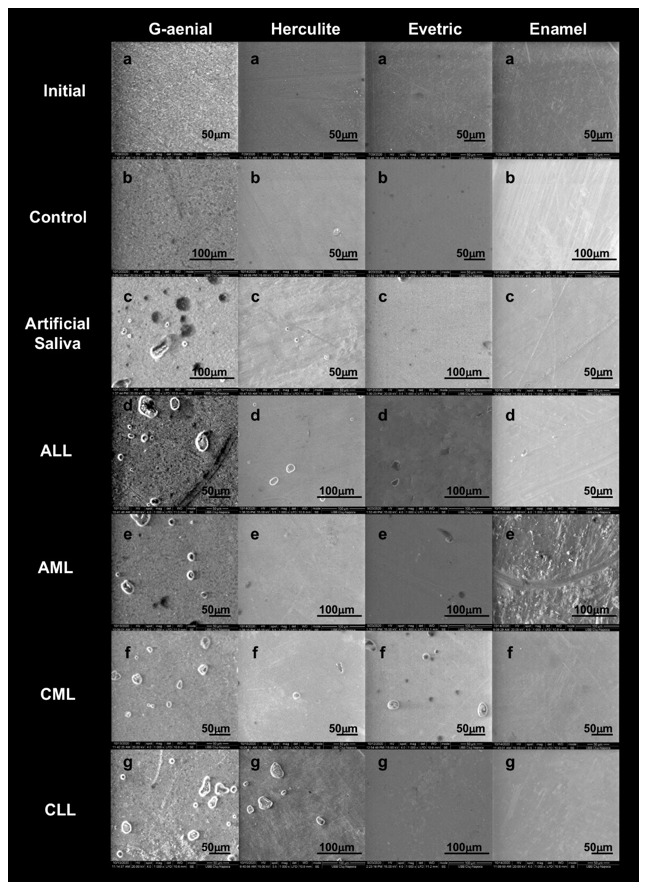
SEM images of RBCs: (**a**) initial (immediately after the polymerization process) and after 7 days of immersion in various saliva: (**b**) control; (**c**) artificial saliva; (**d**) ALL; (**e**) AML; (**f**) CLL; (**g**) CML (1000× magnification).

The SEM figures, prior to sorption testing, shows the presence of surface defects of the initial discs obtained from the process of polymerization and manipulation of the sample. The disc samples after polymerization and immersion in different environments for 7 days indicates in addition to surface defects, the presence of dents and fractures following the process of sorption and solubility. There was a progressive loss of organic matrix with large voids, and a greater exposure of the filler particles from the groups of G-aenial RBC (Figure 4c–g) compared to the Initial and Control group (Figure 4a,b). Gradually, saliva managed to penetrate, causing particles to detach from the surface of the RBC causing voids up to 30 μm in ALL (Figure 4d). Enamel RBC showed less erosion on the surfaces after contact with saliva, without gaps, and with the same morphology of the surface as the initial sample without immersion.

## 4. Discussion

Xerostomia, ulceration and increased dental caries are due to the change in salivary flow and its ability to buffer [11,12]. Oxidative stress can play an important role in malignancies that lead to the development of inflammatory oral pathologies [13]. Saliva is the first line of defence against oxidative stress mediated by free radicals [14]. Besides oxidative stress, endoplasmic reticulum stress has also emerged to play a pivotal role in leukemia and oral cancer, as suggested by Ausiello and Treglia in their research [7,8].

Water sorption and solubility of RBCs are one of the most important problems deteriorating their physical, chemical and mechanical properties [15]. The behaviour of RBCs in different immersion environments varies according to the characteristics of the RBC. Previous studies have shown that filler particles, matrix and binding agents can alter their absorption values [16]. Water sorption values may vary depending on the content and quantity of dilution monomer (such as UDMA or TEGMA), because the ethylene glycol groups, in their composition, are hydrolytic aliphatic groups. The hydrofil monomer content of the RBC may influence the speed and degree of water sorption. From the RBCs tested, Evetric and Herculite do not include UDMA, which are known to be more hydrophobic than Bis-GMA and TEGDMA [17,18]. Bis-GMA monomer has the lowest degree of sorption because it is a large-mass molecule that does not dissolve very quickly in water. However, the type and quantity of monomer is not the only dominant factor deteriorating the degree of sorption and solubility. Its values can also be related to the quantity and the structure of the filler particles and the structure of the binding agent between the two phases that makes up the RBC [19]. 

Ferracane mentioned that RBCs having in its composition silica or quartz-based fillers are considered inert in water [20]. The sorption values of investigated RBCs that have a silica composition (G-aenial and Enamel) are different due to the reduced amount of filling of smaller filler particles (0.04 μ) that can easily be dispersed in the polymer matrix, increasing the degree of conversion and implicitly, so the sorption values are lower. The lowest sorption value of Enamel RBC can also be explained by the increased amount of flexible monomers, which create a dense polymer structure so that the penetration of water molecules is minimal.

The solubility behaviour of the RBCs investigated is also deteriorated by the types of organic matrix and filler. The negative values obtained, for all RBCs investigated, suggest that they are more susceptible to sorption because the weight gain of the samples can mask their actual solubility. Fabre and co-workers have mentioned that this fact could be explained by the hydrophilicity of the organic matrix [21]. Solubility is also related to the amount of non-reactionary monomers, which did not participate in the polymerization reaction of the RBCs. A high degree of conversion leads to a small amount of residual monomer and implicitly to a reduction in solubility. 

Ideally, RBCs should be insoluble and, from a chemical and physical perspective, are stable materials; but their composition prevents this so that most monomers used in dental materials can absorb water and chemicals from the environment and also release components into the oral environment (unactioned monomers). For this determination of the water sorption, the amount of residual monomer released and the integrity of the dental material used are characteristics necessary to determine the degree of degradation of a dental composite. 

In this research, we investigated the influence of saliva on RBCs depending on the type of leukemia. RBCs may undergo chemical, physical and mechanical changes in their properties that leads to a high degree of biodegradation. The most common leachable, potentially toxic agents are monomers not reactivated in the polymerization process, which can induce biological responses to cells and tissues. Degradation of dental materials is influenced by multiple factors such as saliva, oral microbes and the chewing process [22]. Saliva’s, composition may contribute significantly to the biodegradation of acrylic resins. Water molecules can easily penetrate the polymer network allowing the diffusion of unpolymerized monomers and/or additives from the material network [23,24]. 

In addition to the high water content, saliva also contains composition microelements, dissolved salts, enzymes, proteins, amino acids, vitamins, mucus, as well as other substances, which can influence the chemical degradation of dental materials through two mechanisms: hydrolysis and enzyme reaction [25]. Salivary enzymes can degrade polymers by attacking lateral chains, producing both potentially harmful by-products and a deterioration of network properties. Different esterase’s that have been shown to be present in saliva can promote the esterification of methacrylate’s [26,27].

Another important factor that may influence the amount of saliva production is the administration of chemotherapeutic agents and their side-effects that may induce oral dysfunctions. As mentioned by several guidelines, common oral manifestations of leukemia patients following chemotherapy are hyposalivation, xerostomia and salivary gland disfunctions, which may worsen the health status of frail patients if dental care is not performed [1,2,3,4]. Moreover, RBCs used for the dental treatments may suffer destructions and alterations in patients undergoing cytotoxic treatments or bone marrow transplantation, mostly due to the saliva composition. 

Our findings showed that Enamel RBC had the greatest stability, the lowest degree of absorption and a compact, fissure-free structure, in all 7 saliva samples. Regarding the immersion environment, if we compare all three methods of investigation of the 4 RBCs, the most severe damage could be seen in the ALL sample. The CML sample was the least aggressive in terms of degradation of RBCs, after the control and artificial saliva samples. 

The release of residual monomers in the oral environment may influence the structural stability, mechanical properties and the durability of the materials used, as well as their biocompatibility. One of the factors responsible for the release of residual monomers may be the composition of the solvent [28,29]. It has been reported that the dilution monomers, which have a low molecular weight and a higher mobility, can be extracted in larger quantities from the dental composites [29,30,31]. This behaviour was also found in our investigated RBCs, it was found to be less in the AML, ALL and CML saliva samples where the amount of residual UDMA is double than TEGDMA. The smallest amount of residual monomer was for Bis-GMA with the highest mass, being also the most rigid with a quantity of up to 5 times less unreactive and released monomer. Chromatograms (Figure 3, Table 5) showed that the highest quantity of residual monomers extracted from the investigated RBCs was recorded in the AML environment, in which the amounts of sorption and solubility is also higher than the control environment (supported by SEM investigations).

Our research represents an original initiative which may support future studies regarding the use of dental materials in patients with leukemia. However, limits of our paper should be addressed. The relative low number of dental composites that have been used, the in vitro characteristics of the saliva samples and lack of other research available for comparison may serve as a starting point for future studies. Additionally, we were not able to study the influence of leukemia saliva on RBCs during and after chemotherapeutic regimen. Another limitation consists of the inability to study salivary biomarkers whose deficit may lead to oral inflammation and determine an oral cancer. Several authors have mentioned that an early detection of salivary cytokines may contribute to the pathogenesis of oral cancer; it is known that leukemia patients have a high prevalence of developing oral squamous cell carcinoma [2,32,33,34]. 

## 5. Conclusions

Our findings showed that acute leukemia influenced the most RBCs by changing their chemical, physical and mechanical properties compared to chronic leukemia. The composition of RBCs and the saliva environment to which they were exposed have the ability to promote the biodegradation of samples. Additionally, an increase in the percentage of sorption and solubility and the exposure of the filling of materials that cause superficial gaps observed through SEM images was shown. For residual monomers and unreacted monomers, high values were recorded in acute leukemia environments.

## Figures and Tables

**Table 1:** Brand, manufacturer, composition of the RBCs brands tested.

Composite	Resins	Fillers
G-aenial Anterior A2,GC corporation, Tokyo, Japan	UDMA, dimethacrylate co-monomers (Bis-GMA) (37%)	Pre-polymerized fillers containing silica (19–17 µ), pre-polymerized particles containing strontium (400 nm) and lanthanoid fluoride (100 nm), silica (16 nm), fumed silica (63%)
Herculite XRV Ultra A2; Kerr; Italy	Bis-GMA, TEGDMA (41%)	Al-B-Si glass, SiO_2_ (59% by volume, particles 0.6 microns)
Evetric filling material A2, Ivoclar Vivadent	Dimethacrylates (19–20%)	Barium glass, ytterbium trifluoride, mixed oxide, copolymers (80–81 wt.%; 55–57 vol.%, size 40–3000 nm)), additives, catalysts, stabilizers, pigments (80%)
Enamel Plus HRi, UD2, Micerium	UDMA, BisGMA, 1,4-butandiol-dimethacrylate (45%)	Glass filler (0.7 µ), highly dispersed silicone dioxide (0.04 µ) (55%)

**Table 2:** Differences between the experimental conditions within each day, material used and measurement used.

Material	Day	Measured	*p*-Value	Measured	*p*-Value
Gaenial	Day1	Water Sorption	0.708	Water Solubility	0.632
Gaenial	Day2	Water Sorption	0.174	Water Solubility	0.098
Gaenial	Day3	Water Sorption	0.188	Water Solubility	0.012
Gaenial	Day7	Water Sorption	0.065	Water Solubility	0.304
Herculite	Day1	Water Sorption	0.315	Water Solubility	0.467
Herculite	Day2	Water Sorption	0.404	Water Solubility	0.184
Herculite	Day3	Water Sorption	0.154	Water Solubility	0.218
Herculite	Day7	Water Sorption	0.199	Water Solubility	0.132
Evetric	Day1	Water Sorption	0.073	Water Solubility	0.060
Evetric	Day2	Water Sorption	0.415	Water Solubility	0.060
Evetric	Day3	Water Sorption	0.160	Water Solubility	0.131
Evetric	Day7	Water Sorption	0.904	Water Solubility	0.451
Enamel	Day1	Water Sorption	0.669	Water Solubility	0.481
Enamel	Day2	Water Sorption	0.705	Water Solubility	0.129
Enamel	Day3	Water Sorption	0.133	Water Solubility	0.005
Enamel	Day7	Water Sorption	0.680	Water Solubility	0.809

**Table 3:** Pairwise comparison between materials in each day. NA = not applicable.

Variable	Day	Measured	Enamel	Evetric	G-aenial
Evetric	Day 1	Water Sorption	0.623	NA	NA
Gaenial	Day 1	Water Sorption	<0.001	<0.0001	NA
Herculite	Day 1	Water Sorption	0.079	0.123	<0.0001
Evetric	Day 2	Water Sorption	0.102	NA	NA
G-aenial	Day 2	Water Sorption	<0.0001	<0.0001	NA
Herculite	Day 2	Water Sorption	<0.01	0.025	<0.0001
Evetric	Day 3	Water Sorption	<0.001	NA	NA
G-aenial	Day 3	Water Sorption	<0.0001	<0.0001	NA
Herculite	Day 3	Water Sorption	<0.001	0.709	<0.0001
Evetric	Day 7	Water Sorption	<0.01	NA	NA
G-aenial	Day 7	Water Sorption	<0.0001	<0.0001	NA
Herculite	Day 7	Water Sorption	0.014	0.411	<0.0001
Evetric	Day 1	Water Solubility	1	NA	NA
G-aenial	Day 1	Water Solubility	<0.0001	<0.0001	NA
Herculite	Day 1	Water Solubility	0.68	0.382	<0.0001
Evetric	Day 2	Water Solubility	<0.01	NA	NA
G-aenial	Day 2	Water Solubility	<0.0001	<0.0001	NA
Herculite	Day 2	Water Solubility	0.012	0.031	<0.0001
Evetric	Day 3	Water Solubility	<0.01	NA	NA
G-aenial	Day 3	Water Solubility	<0.0001	<0.0001	NA
Herculite	Day 3	Water Solubility	0.011	0.773	<0.0001
Evetric	Day 7	Water Solubility	<0.001	NA	NA
G-aenial	Day 7	Water Solubility	<0.0001	<0.0001	NA
Herculite	Day 7	Water Solubility	0.012	1	<0.0001

**Table 4:** Progression of each material through the assessment days.

Material	Measured	*p*-Value	Tau
G-aenial	Water Sorption	<0.0001	0.58
Herculite	Water Sorption	<0.0001	0.44
Evetric	Water Sorption	<0.0001	0.56
Enamel	Water Sorption	<0.01	0.36
G-aenial	Water Solubility	<0.0001	−0.65
Herculite	Water Solubility	<0.0001	−0.54
Evetric	Water Solubility	<0.0001	−0.73
Enamel	Water Solubility	<0.0001	−0.48

**Table 5:** The amount of monomers released into the samples.

Sample	TEGDMA [%] × 10^−6^	UDMA [%] × 10^−6^	Bis-GMA [%] × 10^−6^	Total Residual Monomer × 10^−6^
Artificial saliva	4.881776707	5.94775482	2.900303374	13.729833488
Control	6.61202044	5.567923011	1.795024772	13.97496822
ALL	13.64720043	5.478786195	2.502141055	21.62812768
AML	16.9537037	29.26157407	4.212962963	50.42824074
CLL	4.609006307	7.747100075	2.355224276	14.71133066
CML	7.051773396	21.27610155	1.435440039	29.76331498

## Data Availability

The data presented in this study are available on request from the corresponding authors.
